# Shock-Resistibility of MEMS-Based Inertial Microswitch under Reverse Directional Ultra-High g Acceleration for IoT Applications

**DOI:** 10.1038/srep45512

**Published:** 2017-03-31

**Authors:** Qiu Xu, Zhuoqing Yang, Yunna Sun, Liyan Lai, Zhiyu Jin, Guifu Ding, Xiaolin Zhao, Jinyuan Yao, Jing Wang

**Affiliations:** 1 National Key Laboratory of Science and Technology on Micro/Nano Fabrication, School of Electronic Information and Electrical Engineering, Shanghai Jiao Tong University, Shanghai, 200240, China; 2 Electrical Engineering Department, University of South Florida, Tampa, FL 33620, USA

## Abstract

This paper presents a novel MEMS-based inertial microswitch design with multi-directional compact constraint structures for improving the shock-resistibility. Its shock-resistibility in the reverse-sensitive direction to ultra-high g acceleration (~hunderds of thousands) is simulated and analyzed. The dynamic response process indicates that in the designed inertial microswitch the proof mass weight *G*, the whole system’s stiffness *k* and the gap *x*_*2*_ between the proof mass and reverse constraint blocks have significant effect on the shock-resistibility. The MEMS inertial microswitch micro-fabricated by surface micromachining has been evaluated using the drop hammer test. The maximum allowable reverse acceleration, which does not cause the spurious trigger, is defined as the reverse acceleration threshold (*a*_*thr*_). Test results show that *a*_*thr*_ increases with the decrease of the gap *x*_*2*_, and the proposed microswitch tends to have a better shock-resistibility under smaller gap. The measured responses of the microswitches with and without constraint structure indicates that the device without constraint structure is prone to spurious trigger, while the designed constraint structures can effectively improve the shock-resistibility. In this paper, the method for improving the shock-resistibility and reducing the spurious trigger has been discussed.

Microelectromechanical systems (MEMS) inertial microswitches based on surface micromachining technology have attracted a great deal of attention due to their small size, lower costs and large volume production^1^,^2^. They are widely used in many applications such as accessories, toys, the transportation of special goods, automotive electronics and remote monitoring (RMON)^3^,^4^,^5^. In the coming years, the rapid growth of internet of things (IoT) will lead to a growing demand of MEMS inertial switches which are used for monitoring in IoT devices. The MEMS inertial microswitch behaves as a passive device that does not consume power until an acceleration event occurs. Therefore, it can be used in some long-lifetime systems such as the internet of things in spite of the limited available power. The new chipless radio frequency identification (RFID) tag can operate at the conventional UHF band for RFID applications. In the presented scheme, MEMS inertial switches are used as programmable elements to support communication between the tag and RFID interrogators^6^,^7^,^8^. It can be employed to monitor the shock from the environment in order to protect the device against the damage from the overload shock in the IoT sensors.

The working principle of an inertial switch is that the movable electrode moves along the sensitive direction and comes into contact with the stationary electrode to form an electric path when it is triggered by an over-threshold acceleration in the sensitive direction. If the microswitch is subjected to a large enough acceleration in the reverse sensitive direction, large deflection of the suspension spring would occur, thus causing the proof mass (i.e., movable electrode) to rebound in the sensitive direction. A spurious trigger may happen if the rebounding distance of the proof mass reaches the gap *x*_*1*_ between the proof mass and stationary electrode. In the previous reports^9^,^10^,^11^,^12^,^13^, several kinds of microswitches have been designed, simulated and fabricated, while the threshold acceleration, the switch-on time and the reliability are discussed when the acceleration is applied in the sensitive direction. In the practical applications, the inertial microswitch will inevitably be subject to high or even ultra-high shocks along the reverse direction because of the complicated working environment. Unfortunately, very few prior works addressed the shock-resistibility issue by reducing the spurious trigger. In this regard, a novel laterally-driven inertial microswitch with multi-directional compact constraint structures is proposed and thoroughly investigated. Moreover, the effects of the proof mass weight *G*, the whole system stiffness *k* and the gap *x*_*2*_ on the shock-resistibility are analyzed when the switch is subjected to an acceleration in the reverse sensitive direction (-*x* direction).

The lowest reverse acceleration, which is the onset critical value for making the switch to turn on incorrectly, is defined as the reverse acceleration threshold (*a*_*th*r_). As a result, the circuit will not conduct current when the applied reverse acceleration is less than *a*_*thr*_. It is a very crucial parameter for the microswitch, which represents the shock-resistibility of the designed device. The proposed inertial microswitch design will be simulated and evaluated when the reverse threshold acceleration has been applied in the simulation model. Finally, the fabricated inertial microswitch has been fully tested by a drop hammer system.

## Results

### Structure design

**Figure 1:**
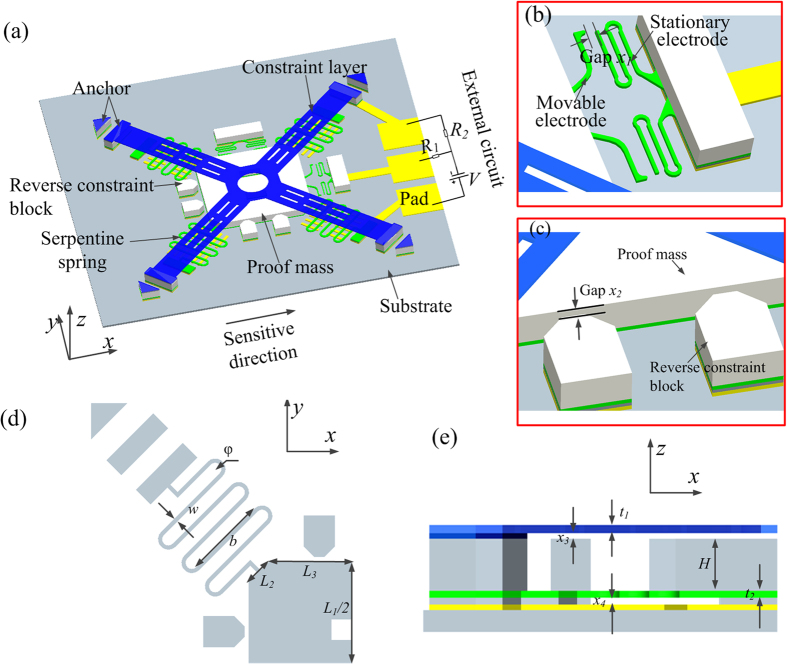
The schematic diagram and structure parameters of the designed inertial microswitch. (**a**) The 3D view of the whole structure. (**b**,**c**) The close-ups of the switch. (**d**) The top view of a quarter of the switch. (**e**) The side view of the one-half structure.

As compared to the silicon substrate, the insulated quartz substrate is less likely to be broken to pieces under the ultra-high g acceleration. When the insulated quartz substrate is heated during the fabrication process, it won’t delaminate from the metal switch microstructures due to the small thermal expansion coefficient of quartz. Therefore, the novel inertial microswitchs with multi-directional compact constraint structures for improving the shock-resistibility have been designed and fabricated over an insulated quartz substrate. The microswitch mainly consists of three parts: two L-shape cantilever beams attached to the proof mass as the movable electrodes; one spring shape cantilever beam supported by anchors as the stationary electrode; and the compact constraint structures consisting of constraint layer with holes and reverse constraint blocks to limit the vibration in the reverse sensitive direction. The schematic diagram of the designed microswtich is shown in Fig. 1(a). The supply voltage source *V*, the bias resistance *R*_*1*_ (300 Ω) and the sufficient switch movement in the *x* direction form an electric path when the two electrodes touch. The supply voltage source *V*, the bias resistance *R*_*2*_ (300 Ω) and the sufficient switch movement in the *z* direction make another circuit when the proof mass collides with the constraint layer. In this designed inertial microswitch, the two sensitive in-plane directions of the device (*x* direction and *y* direction) are equivalent and they are parallel to the substrate plane. In this paper, the *x* direction characteristics will be mainly discussed. The gap *x*_*1*_ between movable electrode and stationary electrode has been set to 20 μm as shown in Fig. 1(b). The gap *x*_2_ between the proof mass and reverse constraint block is depicted in Fig. 1(c). Figure 1(d,e) show the top view and the side view of the microswitch, respectively.

### Physical model and theoretical analysis

**Figure 2:**
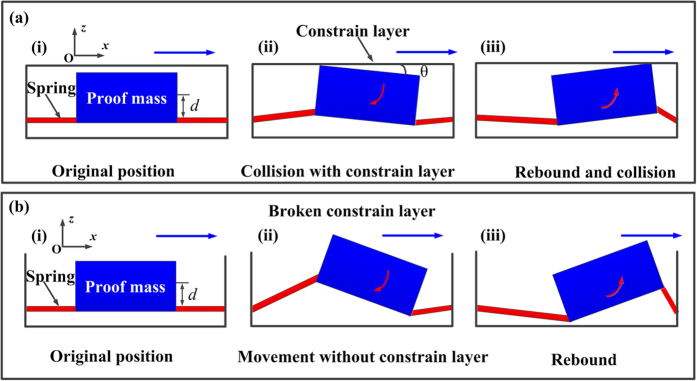
Rotation and movement process of the proof mass in moving toward stationary electrode (**a**) with constraint layer and (**b**) without constraint layer.

The inertial microswitch with the constraint layer can be simplified to a spring-mass system with constraint structures as shown in Fig. 2(a). (i) Firstly, the proof mass will rebound toward the stationary electrode after it collides with the reverse constraint block. (ii) Considering that the centroid deviation between the proof mass and serpentine springs inevitably exists during the resilient process, the caused moment will make the proof mass rotate in clockwise direction in the *xoz* plane until it collides with the upper constraint layer. (iii) The proof mass will also rotate in the anticlockwise direction under the action of collision rebound. Finally, the proof mass may collide with constraint structures a few times in moving toward stationary electrode. The whole system’s energy will be dissipated to some extent due to the frequent collisions between the proof mass and the top constraint structures. Therefore, the resilient distance in the sensitive direction is reduced. Correspondingly, it is more robust to the spurious trigger that is more difficult to happen. As soon as the constraint layer is broken, the inertial microswitch can be simplified and sketched in Fig. 2(b). If the inertial microswitch rebounds in the sensitive direction, there is no collision between the proof mass and constraint layer. Then, the proof mass will freely rotate in the *xz* plane. The whole system’s energy will not be easily consumed as compared to the switch with constraint layer. Apparently, the resilient distance without the constraint layer in the sensitive direction is larger than the one with the constraint layer. The device without the constraint layer is much more prone to spurious trigger.

In this paper, the delay from time zero (zero input) to the time when the proof mass firstly starts to contact the constraint layer is defined as the collision response time (*t*_*c*_) in *z*-axis direction. The first collision response time is related to the off-axis sensitivity of inertial microswitch. When the microswitch with constraint structures is triggered by the acceleration, the first collision response time decreases with the increases of the collision frequency between the proof mass and constraint layer. Correspondingly, the whole system’s energy is dissipated to some extent due to the frequent collision. In order to better understand the dynamic behavior of designed inertial microswitch, an analytical method is proposed to evaluate the collision response time (*t*_*c*_).

It can be seen from Fig. 2(a) that the proof mass collides with constraint layer when the proof mass rotates by a *θ* angle in clockwise direction of the *xz* plane. The rotation of proof mass can be expressed as follows:


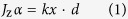


where *J*_*z*_ represents the rotational inertia of system, *α* stands for the angular acceleration, *x* is the displacement of proof mass, and *d* represents the distance between centroid of proof mass and the plane of serpentine springs.

When the inertial microswitch is mechanically shocked by a reverse acceleration at the threshold value *a*_*thr*_, the proof mass firstly collides with the reverse constraint block and then rebounds in the sensitive direction. In this paper, this rebound acceleration is equivalent to a sensitive acceleration *a* applied to the device. In practice, the acceleration *a* is usually a half-sine wave with the peak value *a*_*0*_ and frequency *w*_*0*_. During this rebound process in the sensitive direction, the differential equation of motion can be expressed as follows:


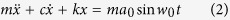


where *m* is the mass of proof mass, *c* is the damping coefficient, *k* represents the whole system spring constant of four constituent serpentine springs, *x* is the displacement of proof mass.

**Figure 3:**
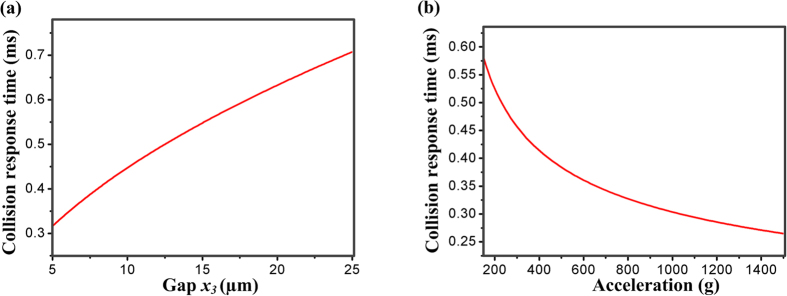
Collision response time between the proof mass and constraint structures changing with (**a**) gap (*x*_*3*_) between the proof mass and constraint structures and (**b**) applied acceleration amplitude in the sensitive direction.

By solving the Eqs (1) and (2)^ ^14, the first collision response time (*t*_*c*_) between the proof mass and constraint structures in *z*-direction can be expressed as:


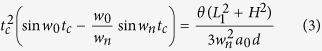


where, *L*_1_ and *H* are length and height of the proof mass, respectively, *ω*_n_ is the inherent frequency of mass-spring inertial system and *θ* is the rotation angle, as shown in Fig. 2(a). It can be seen from Fig. 2(a) that the ration angle *θ* can be expressed as follows:


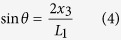


where *x*_*3*_ is the gap between proof mass and constraint layer. According to Eq. (3), one can obtain plots of the first collision response time varied with applied acceleration amplitude and gap (*x*_*3*_) between proof mass and constraint layers. It can be observed in Fig. 3(a) that the collision response time increases with the gap (*x*_*3*_) between proof mass and constraint structures. Figure 3(b) shows that the collision response time decreases with the increase of the rebound acceleration *a*. The collision response time reflects the frequency at which the proof mass collides with the constraint layer. The shorter the collision response time is, the more frequently the proof mass would collide with the constraint structures. Therefore, the resilient distance in the sensing direction becomes smaller because the whole system energy is dissipated to some extent. The movement of the proof mass should be restrained and the spurious trigger should not happen easily because of the frequent collision between the proof mass and the constraint layer. As a result, the shock-resistibility of the microswitch is improved. When the constraint layer is broken, the spurious trigger easily occurs due to the lack of energy-dissipating collision events with the constrain layer.

## Methods

### FEM simulation

The ANSYS software was employed to simulate the dynamic contact process. A convenient and accurate analysis can be conducted by this finite element method (FEM) software to evaluate the resistance threshold to the reverse accelerations when the proof mass rebounds in the sensitive direction. From the previous experimental results^15^, the main structural material of the inertial switch is electroplated nickel and its properties are as follows: Poisson’s ratio is 0.3, Young’s modulus is 171.5 GPa and density is 8.96 g · cm^−3^.

In the practical applications, the inertial microswitch will inevitably be subject to shocks from different directions. Especially in the opposite direction, the spurious trigger will happen if the resilient distance of the movable electrode is exactly equal to the gap *x*_*1*_ (20 μm) between the stationary electrode and the movable electrode. Therefore, the dynamic response of inertial microswitch mechanically shocked in the reverse sensitive direction should be investigated. The maximum allowable reverse acceleration, which does not cause the switch spurious trigger, is defined as the reverse acceleration threshold (*a*_*thr*_). The acceleration *a*_*thr*_ indicates the shock-resistibility of the inertial switch and it is the significant parameter for evaluating the structure of designed inertial microswitch. For an inertial switch, the key factors affecting *a*_*thr*_ are the proof mass weight *G*, the whole system stiffness *k* and the gap *x*_*2*_ between proof mass and reverse constraint block. In the following section, the three factors will be analyzed individually.

### Effect of the proof mass weight *G* on the shock-resistibility of inertial microswitch

**Figure 4:**
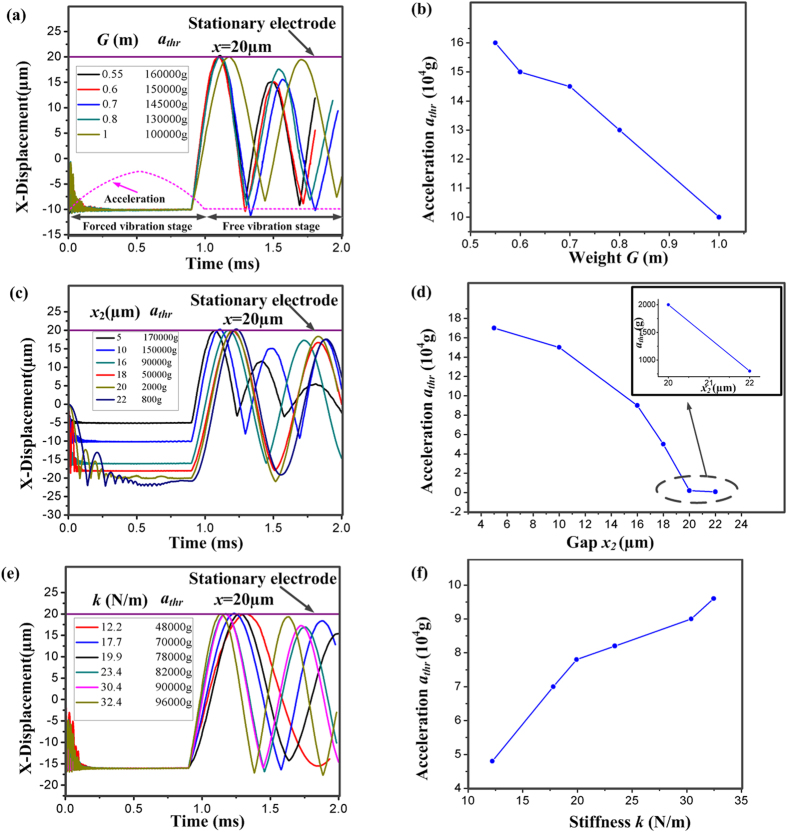
The dynamic response curves of movable electrode under the maximum allowable reverse acceleration threshold *a*_*thr*_ when (**a**) different proof mass weight *G*, (**c**) different gap *x*_*2*_ between the proof mass and reverse constraint block; (**e**) different whole system stiffness *k*. Dependences of *a*_*thr*_ on (**b**), *G* (**d**) *x*_*2*_ and (**f**) *k*.

The proof mass weight *G* is one of the most important factors influencing the reverse acceleration threshold *a*_*thr*_. Here, the thickness *t*_*0*_ of the spring and the gap *x*_*2*_ are set to 10 μm. As a result, the whole system stiffness *k* and the gap *x*_*2*_ are invariant. The proof mass weight *G* is defined as ‘*m*’ when the thickness *H* of proof mass is 100 μm. If the thickness *H* of proof mass is 55 μm, 60 μm, 70 μm and 80 μm, correspondingly the proof mass weight *G* is 0.55 *m*, 0.6 *m*, 0.7 *m* and 0.80 *m* due to the same in-plane geometrical structure. Figure 4(a) shows the simulated displacement-time curves of movable electrode under the maximum allowable reverse acceleration *a*_*thr*_ for different varied proof mass weight *G*. When the *G* changes from 0.55 m to m, the corresponding reverse acceleration threshold *a*_*thr*_ is 160000 g, 150000 g, 145000 g, 130000 g and 100000 g, respectively. Here, the acceleration of 1 g is equal to 9.8 m/s^2^. The pulse width is 1 ms. It can be seen from Fig. 4(b) that the reverse acceleration threshold *a*_*thr*_ decreases with the increase of the proof mass weight *G*. In other words, the microswitch tends to have better shock-resistibility under smaller *G*. Figure 4(a) indicates that the proof mass firstly moves in the reverse sensitive direction and collides with the reverse constraint block during the forced vibration process. Then it rebounds and the spurious trigger occurs during the free vibration process.

### Effect of the gap *x*_*2*_ on the reverse acceleration threshold *a*_*thr*_

In addition, the gap *x*_*2*_ between proof mass and reverse constraint block also has a great impact on the reverse acceleration threshold *a*_*thr*_. Figure 4(c) shows the dynamic response curves of movable electrode under the maximum allowable reverse acceleration *a*_*thr*_ for different varied gap *x*_*2*_. Here, the thickness of the spring and the proof mass is 10 μm and 80 μm, respectively. When the gap *x*_*2*_ changes from 5 μm to 22 μm (i.e. 5 μm, 10 μm, 16 μm, 18 μm, 20 μm and 22 μm), the reverse acceleration threshold *a*_*thr*_ is 170000 g, 150000 g, 90000 g, 50000 g, 2000 g and 800 g, respectively. It can be observed from Fig. 4(d) that *a*_*thr*_ decreases with the increase of the gap *x*_*2*_ between the proof mass and reverse constraint block. As a consequence, the microswitch tends to have better shock-resistibility under smaller gap *x*_*2*_. For the inertial switch, reduction of the gap *x*_*2*_ is an effective way to circumvent the spurious trigger and make the device more reliable.

### Effect of the whole system stiffness *k* on the reverse acceleration threshold *a*_*thr*_

In order to investigate the dependence of the shock-resistibility of inertial microswitch on the whole system stiffness *k*, the invariant gap *x*_*2*_ and the proof mass weight *G* are set as 16 μm and 0.8 m, respectively. Figure 4(e) shows the displacement-time curves of movable electrode for different the whole system stiffness *k*. When the thickness *t*_*0*_ of the spring changes from 4 μm to 11 μm (i.e. 4 μm, 6 μm, 7 μm, 8 μm, 10 μm and 11 μm), the corresponding whole system stiffness *k* is 12.21 N/m, 17.79 N/m, 19.92 N/m, 23.42 N/m, 30.41 N/m and 32.46 N/m, respectively. The reverse acceleration threshold *a*_*thr*_ is 48000 g, 70000 g, 78000 g, 82000 g, 90000 g and 96000 g, respectively. It can be seen from Fig. 4(f) that *a*_*thr*_ increases with the whole system stiffness *k*. As a result, the microswitch tends to have better shock-resistibility under larger stiffness *k*.

### Effect of constraint structures on the reverse acceleration threshold *a*_*thr*_

**Figure 5:**
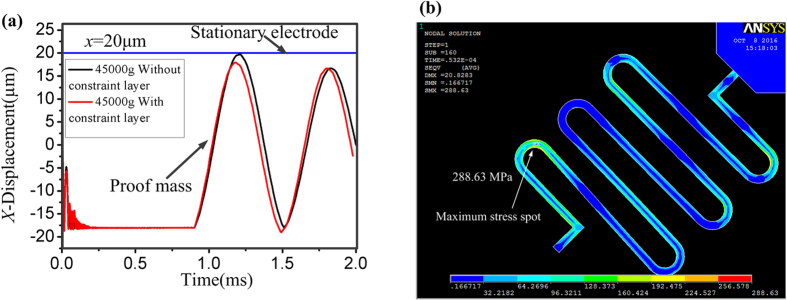
(**a**) The dynamic response curves of movable electrode with constraint layer and without constraint layer under the same acceleration 45000 g in the reverse sensitive direction. (**b**) Stress distribution of the serpentine spring reaching the reverse maximum displacement under the reverse acceleration of 45000 g.

In the practical applications, the inertial microswitch will inevitably be subject to shocks in different directions because of the complicated working environment. Especially in the reverse sensitive direction, the possible spurious trigger will happen if the resilient distance reaches the gap *x*_*2*_ between the proof mass and the reverse constrain block. Therefore, the dynamic response process of the inertial microswitch triggered by the reverse sensitive acceleration should be investigated. In this design, a constraint layer is introduced to restrain the overload impaction from the opposite direction. Figure 5(a) shows the dynamic responses curves of movable electrode with and without the constraint layer. It indicates that the resilient distance with the constrain layer is less than the gap *x*_*2*_ while the resilient distance without the constrain layer is equal to the gap *x*_*2*_ under the same reverse sensitive acceleration of 45000 g. As a result, the spurious trigger will occur if the constraint layer is broken. Therefore, the constraint layer can improve the shock-resistibility of the inertial microswitch and eliminate the spurious trigger. When the inertial switch is mechanically shocked by the applied acceleration of 45000 g in the reverse direction, the maximum stress of the serpentine spring reaching the reverse maximum displacement is 288.63 MPa as shown in Fig. 5(b), which is smaller than the yield strength of electroplated nickel of 317 MPa.

In short, the reverse acceleration threshold *a*_*thr*_ decreases with the increase of the proof mass weight *G* and the gap *x*_*2*_, and *a*_*thr*_ increases with the whole system stiffness *k*. Besides, the constraint layer can improve the shock-resistibility and prevent the spurious trigger.

### Device Micro-Fabrication

**Figure 6:**
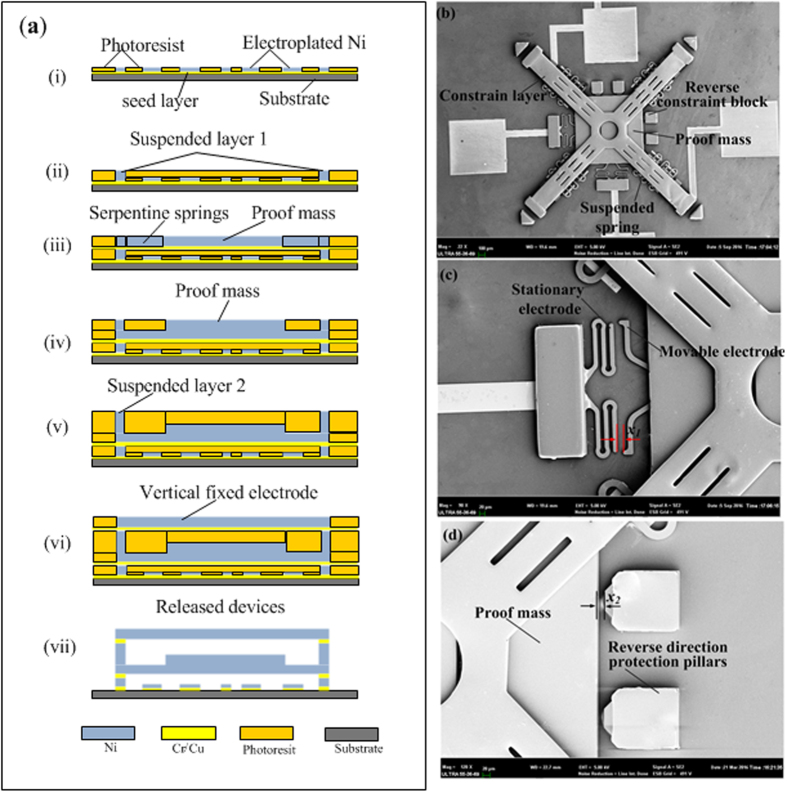
(**a**) The main fabrication process of the inertial microswitch; (**b**) The SEM micrographs of the fabricated inertial microswitch; (**c**,**d**) Its close-ups.

The designed inertial microswitch was fabricated on the quartz substrate by a multilayer lithography and electroplating process based on the surface micromachining technology. The main fabrication process steps of the inertial microswitch is illustrated in Fig. 6(a) and described as follows:

- Firstly, Cr/Cu with a 300 nm total thickness was sputtered on the quartz wafer substrate. Then an array of the pads, anchors and raised strips were electroplated in nickel after the photoresist mold was patterned.
- Next, the anchors, which suspended the springs, were electroplated for *x*_*4*_ higher than the anti-adhesion strips in order to form the first suspended layer.
- The spring layer was electroplated after the Cr/Cu was sputtered on the first suspended layer as the second seed layer.
- The proof mass was electroplated up to the required thickness by multiple lithography and multi-layer electroplating process technology.
- The anchors were fabricated thicker than the proof mass in order to build a gap *x*_*3*_ between the bottom of constraint layer and the top of proof mass.
- The constraint layer was electroplated after lithography technology.
- The photoresist and the chromium/copper seed layer were completely removed by sodium hydroxide solution and an ammonia/peroxide solution, respectively.

Figure 6(b) is the SEM micrograph of the released, completed inertial microswitch. Figure 6(c) shows the close-up image of the gap *x*_*1*_ between the movable electrode and the stationary electrode, while Fig. 6(d) present close-up photo of the gap *x*_*2*_ between the proof mass and the reverse constraint block.

## Discussions

### Experiment test

**Figure 7:**
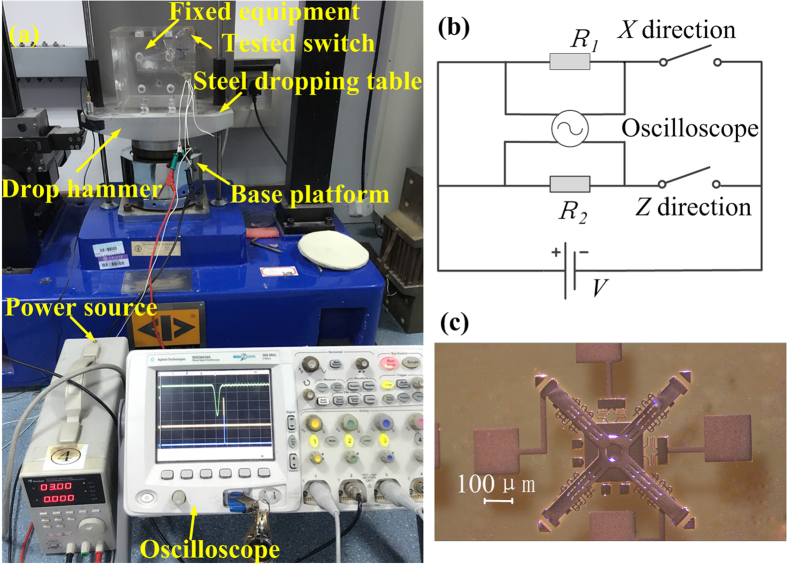
(**a**) Dropping shock equipment for testing the fabricated inertial microswitch; (**b**) The schematic diagram of test circuit; (**c**) The photo of the fabricated inertial microswitch.

As shown in Fig. 7(a), the fabricated inertial microswitches were characterized by a drop hammer test. The drop hammer was freely dropped onto the base platform from different pre-determined height *H*. The amplitude of acceleration increases with the height *H*. The test circuit is shown in Fig. 7(b). If the inertial microswitch was shocked by the acceleration, which was larger than its threshold level, the spurious trigger signal (yellow signal), *z*-off-axis collision trigger signal (purple signal) and accelerometer signal (green signal) could be simultaneously captured by the multi-channel oscilloscope.

### Effect of the gap x_2_ on the test acceleration a_thr_

**Figure 8:**
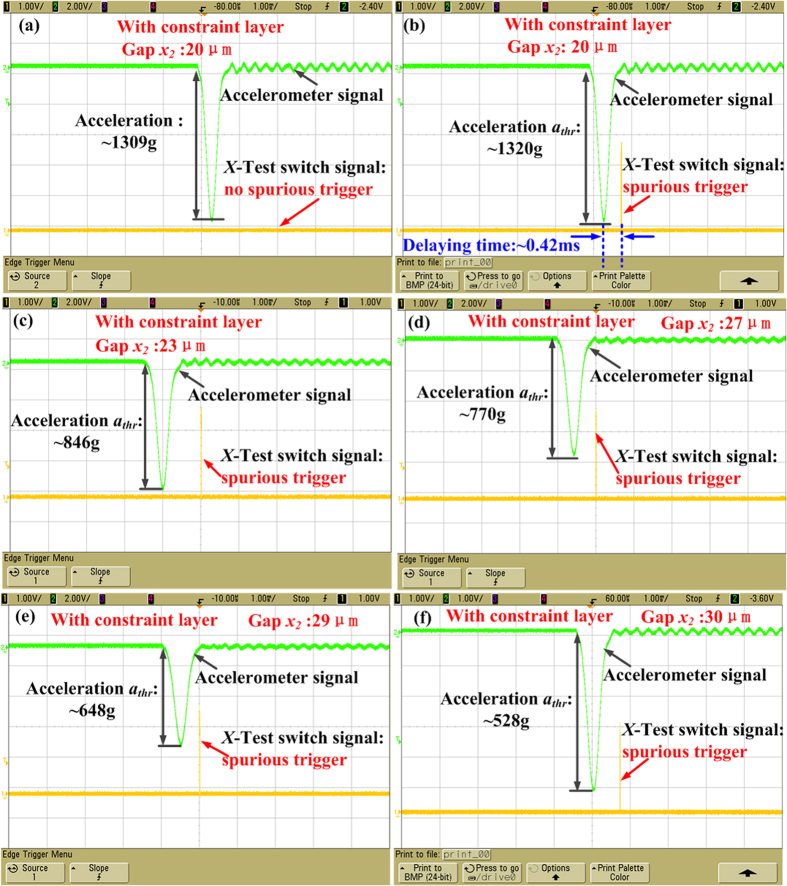
Test results of the fabricated inertial switch. (**a**) The test switch is not triggered under the reverse acceleration 1309 g. (**b**–**f**) Test reverse acceleration threshold *a*_*thr*_ (1320 g, 846 g, 770 g, 648 g, 528 g) of the fabricated inertial microswitch with constraint layer when the gap *x*_*2*_ between proof mass and reverse constraint block is 20 μm, 23 μm, 27 μm, 29 μm and 30 μm, respectively.

**Figure 9:**
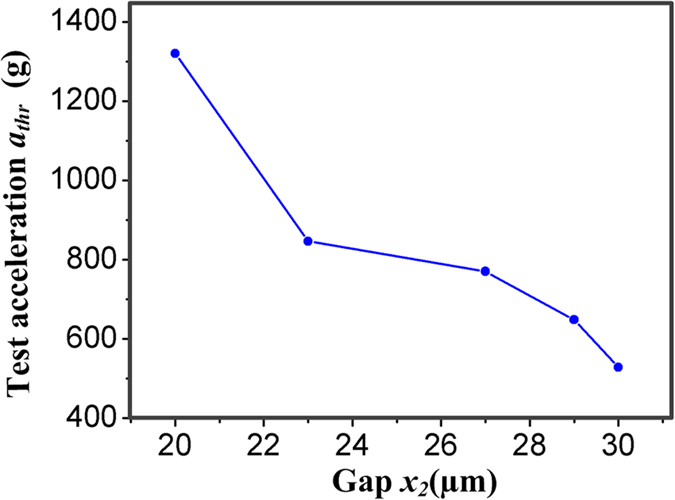
Dependence of tested reverse acceleration threshold *a*_*thr*_ on the gap *x*_*2*_ between proof mass and reverse constraint block.

The test results of the fabricated inertial microswitches with different acceleration peak values are presented in Fig. 8. When the drop hammer was dropped from a height of about 210 cm above the base platform, the fabricated inertial microswitch experienced an acceleration of 1309 g in the reverse sensitive direction (-*x* direction). It can be seen from Fig. 8(a) that there is no trigger signal observed in the sensitive +*x* direction. The height *H* and the acceleration were then increased gradually until an output impulse began to appear, as shown in Fig. 8(b). The minimum applied acceleration in the reverse direction for microswitch to result in a spurious trigger was defined as the reverse acceleration threshold *a*_*thr*_, which was 1320 g for the inertial microswtich of sample 1, whose gap *x*_*2*_ between the proof mass and reverse constraint block is 20 μm. Figure 8(b) indicates that the spurious trigger occurs after the half-sine wave acceleration, the delaying time is about 0.42 ms. The reason is that the rebound of the proof mass appears after the free vibration stage, which is in good accordance with the simulated results. When the gaps *x*_*2*_ between proof mass and reverse constraint block are 23 μm, 27 μm, 29 μm and 30 μm, respectively, the test reverse acceleration threshold values are 846 g, 770 g, 648 g and 528 g, respectively, as shown in Fig. 8(c–f). Figure 9 shows that tested reverse acceleration threshold *a*_*thr*_ decreases with the increase of the gap *x*_*2*_ between proof mass and reverse constraint block. It is demonstrated that the inertial microswitch tends to have better shock-resistibility under smaller gap *x*_*2*_.

**Figure 10:**
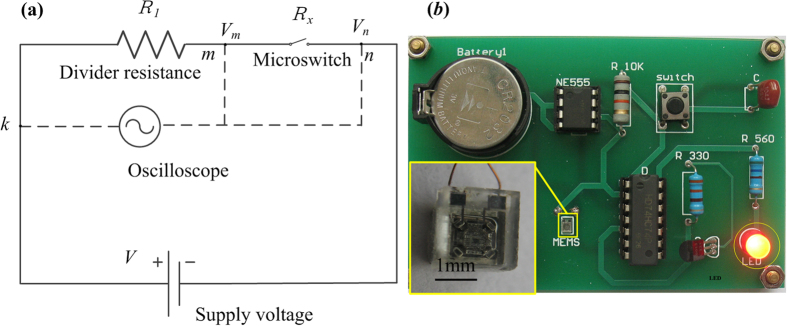
(**a**) The testing principle diagram of the contact resistance. (**b**) Test of the PCB with packaged MEMS inertia microswitch under the reverse acceleration for vibration monitoring systems.

Figure 10(a) shows the schematic for the measurement of the contact resistance in the fabricated microswitch. The divider resistance *R*_1_ (300 Ω), the contact resistance *R*_x_ of the inertial microswitch, copper wires and constant-voltage power supply (7 V) form an test electric path when the movable electrode comes into contact with the stationary electrode under the applied acceleration. In this circuit, the divider voltage (*V*_*m*_) of the divider resistance can be detected by connecting the oscilloscope to point *m*, and the voltage of divider resistance and the switch (*V*_*n*_) can be detected by connecting the oscilloscope to point *n*. The contact resistance *R*_x_ of the inertial microswitch can be obtained according to the law of current conservation in the series circuit:


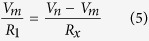


where, *V*_*n*_ = 6.8 V, *V*_*m*_ = 6.6 V and *R*_*1*_ = 300 Ω. The contact resistance is calculated to be 9.09 Ω by the Eq. (5).

There is a tested PCB with packaged MEMS inertial microswitch after undergoing the reverse threshold acceleration. As shown in Fig. 10(b), the LED on the PCB just turns on under the acceleration 1320 g in the reverse sensing direction. The tested reverse acceleration threshold of the MEMS inertial microswitch is 1320 g and the response time is about 0.42 ms. The PCB module with packaged MEMS inertial microswitch can be used as a shock vibration monitoring unit in the wireless sensor network node for IoT applications.

### Effect of the constraint layer on the test acceleration *a*_*thr*_

**Figure 11:**
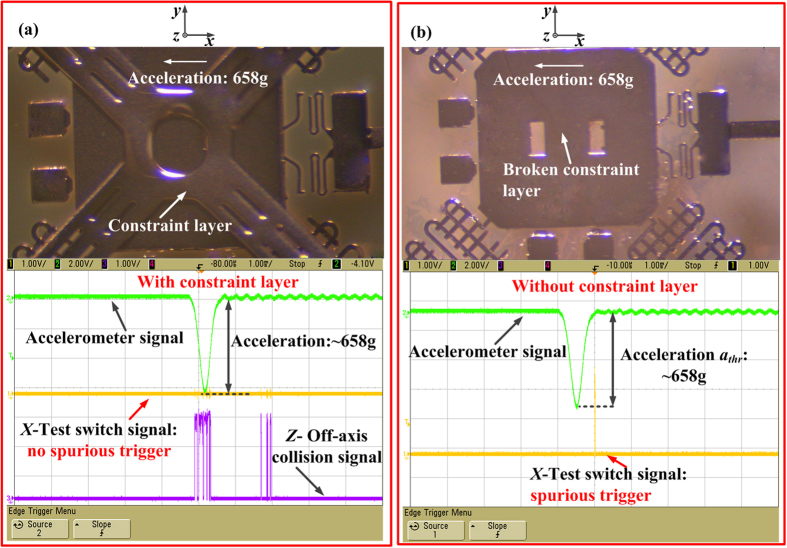
(**a**) The optical photograph of the fabricated microswitch with constraint layer and test results under reverse acceleration 658 g (no spurious trigger). (**b**) The optical photograph of the fabricated microswitch when constrain layer is broken and test result under the same reverse acceleration 658 g (have spurious trigger).

**Figure 12:**
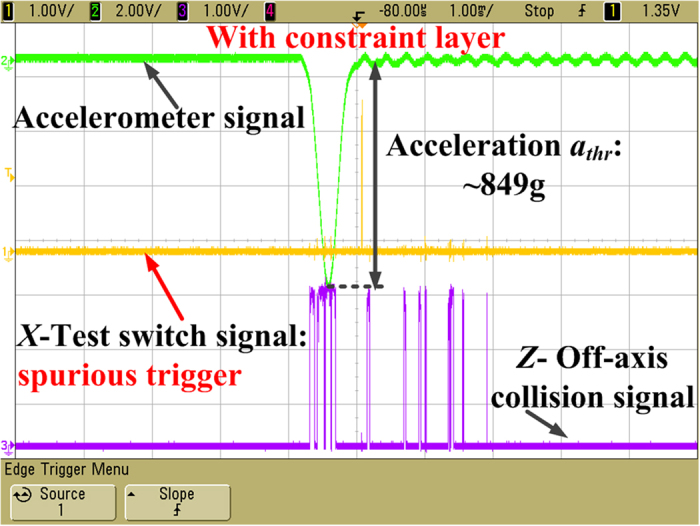
Spurious trigger happens until the reverse acceleration increases up to 849 g (*a*_*thr*_) in the microswitch with constraint layer inFig. 11(a).

In order to investigate the function of the constraint layer, the acceleration of 658 g in the reverse sensitive direction (-*x* direction) has been applied to the fabricated inertial microswitch. Figure 11(a) shows the photo of the fabricated microswitch with constraint layer and the test result under the reverse acceleration of 658 g, which indicates that the spurious trigger in the fabricated switch with the constraint layer is prevented. The constraint layer of previous tested inertial microswitch was broken artificially to validate the impact of the constraint layer. The photo of the fabricated microswitch with a broken constraint layer is shown in Fig. 11(b). When it was shocked by the same acceleration of 658 g in the reverse sensitive direction, the spurious trigger can be readily observed as shown in Fig. 11(b). The characterization and test results demonstrate that the constraint layer in the designed inertial microswitch can restrain the movement of the proof mass and eliminate the spurious trigger. The *z*-off-axis collision as shown in Fig. 11(a) can be observed, which indicates that the proof mass collides with the constraint layer again and again under the reverse sensitive acceleration of 658 g. The whole system energy is consumed to some extent and the resilient distance is reduced which is the reason for the spurious trigger in the inertial microswitch with the constraint layer to be eliminated. It can be seen from Fig. 12 that spurious trigger does not happens until the reverse acceleration increases up to 849 g (*a*_*thr*_) in the microswitch with the constraint layer. It is evident that the reverse resistant threshold acceleration *a*_*thr*_ (658 g) of the microswitch without the constraint layer is smaller than *a*_*thr*_ (849 g) of the inertial microswitch with the constraint layer. Therefore, it is demonstrated that the constraint layer can improve the shock-resistibility.

## Conclusion

A novel inertial microswitch with multi-directional compact constraint structure for improving the shock-resistibility was proposed and demonstrated. Its shock-resistibility in the reverse sensitive direction to ultra-high g acceleration (~hunderds of thounsands) was simulated. The simulation reveals that in the designed inertial microswitch the proof mass weight *G*, the whole system stiffness *k* and the gap *x*_*2*_ between proof mass and reverse constraint blocks are important influential factors for the shock-resistibility in the reverse sensitive direction. The reverse acceleration threshold (*a*_*thr*_) decreases with the increase of the proof mass weight *G* and the gap *x*_*2*_, while increasing with the whole system stiffness *k*. The inertial microswitch fabricated by surface micromachining technology has been evaluated by a standard drop hammer test. The test results show that *a*_*thr*_ increases with the decrease of the gap *x*_*2*_, and the microswitch tends to have better shock-resistibility under smaller gap. The comparison of the measruement results of the microswitches with and without the designed constraint structure indicates that the design without the constraint structure is more susceptible to spurious trigger and the designed constraint structures can effectively improve the shock-resistibility. These conclusions obtained in this work will provide some fundamental guidance for the future design and fabrication of the inertial microswitch in order to further improve shock-resistibility and circumvent the spurious trigger. And the developed passive MEMS inertial microswitch is promising to be used as a shock vibration monitoring sensor for IoT application.

## Additional Information

**How to cite this article:** Xu, Q. *et al*. Shock-Resistibility of MEMS-Based Inertial Microswitch under Reverse Directional Ultra-High g Acceleration for IoT Applications. *Sci. Rep.* **7**, 45512; doi: 10.1038/srep45512 (2017).

**Publisher's note:** Springer Nature remains neutral with regard to jurisdictional claims in published maps and institutional affiliations.
